# Supplementary material for: SOX2+ sustentacular cells are stem cells of the postnatal adrenal medulla
Source: Nat Commun. 2025 Jan 2;16:16. doi: 10.1038/s41467-024-55289-5 (PMC11696870; doi:10.1038/s41467-024-55289-5)
Supplement: Supplementary file 1 — Supplementary Information [file 41467_2024_55289_MOESM1_ESM.pdf]

Supplementary Figure 1

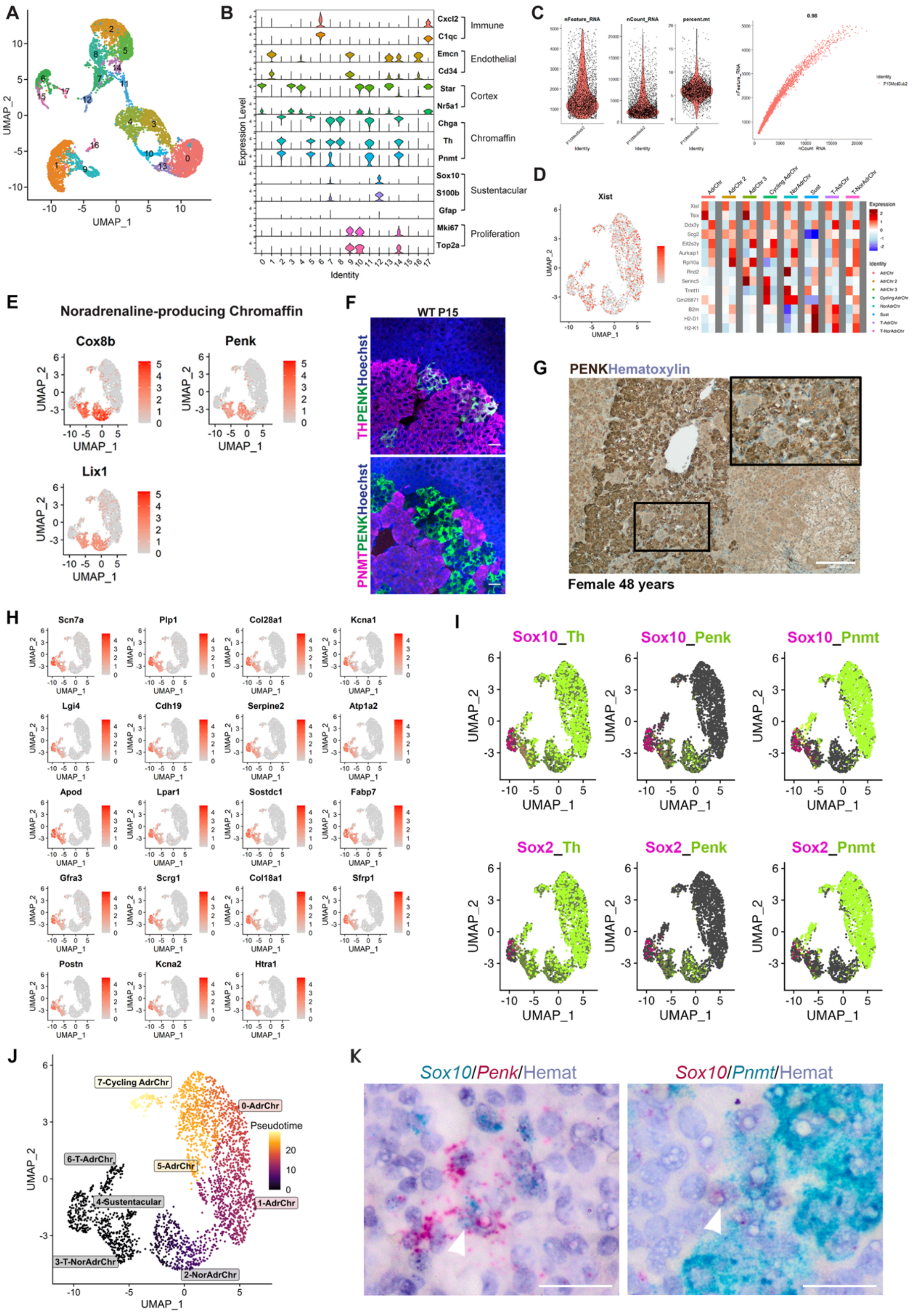

Supplementary Figure 1. Single-cell RNA sequencing of the mouse adrenal medulla. A) UMAP of the entire postnatal medulla dataset obtained (9961 cells); B) Violin plots indicating expression markers chosen for downstream subsetting. C) QC for final dataset used. D) Featureplot for expression of *Xist*, indicating female cells in the dataset. Heatmap of all the differentially expressed genes between sexes in each cluster. E) Featureplots showing newly identified markers *Cox8b*, *Lix1* and *Penk*, specific to the noradrenaline-producing chromaffin cell cluster. F) Immunofluorescence with antibodies against PNMT or TH (magenta) and against PENK (green)on wild type P15 adrenals. Nuclei counterstained with Hoechst, scale bar 20µm. G) Immunohistochemistry on a human adrenal medulla (Female, 48 years of age) using antibodies against PENK (brown) confirming expression. Nuclei counterstained with Hematoxylin, Scale bar 200µm, inset 50µm. H) Featureplots showing newly identified markers of mouse sustentacular cells. I) Featureplots showing gene expression of *Sox2* or *Sox10* with differentiated cell markers *Th*, *Penk*, *Pnmt*. J) Monocle pseudotime UMAP. Sust- sustentacular cells; NorAdrChr Noradrenaline chromaffin lineage; AdrChr Adrenaline chromaffin lineage; Cycling AdrChr cycling adrenaline chromaffin cells, T-AdrChr Transitioning adrenaline chromaffin lineage, T-NorAdrChr Transitioning noradrenaline chromaffin lineage. K) RNAscope mRNA in situ hybridisation on P14 adrenals, using probes against *Sox10* (cyan, sustentacular) and *Penk* (red, noradrenaline chromaffin), or *Sox10* (red, sustentacular) and *Pnmt* (cyan, adrenaline chromaffin), showing rare double-positive cells (arrowheads). Nuclei counterstained with hematoxylin. Scale bars 20µm. For featureplots in D, E and H, colour scale represents Log-normalised expression level, from low (grey) to high (red).

Supplementary Figure 2

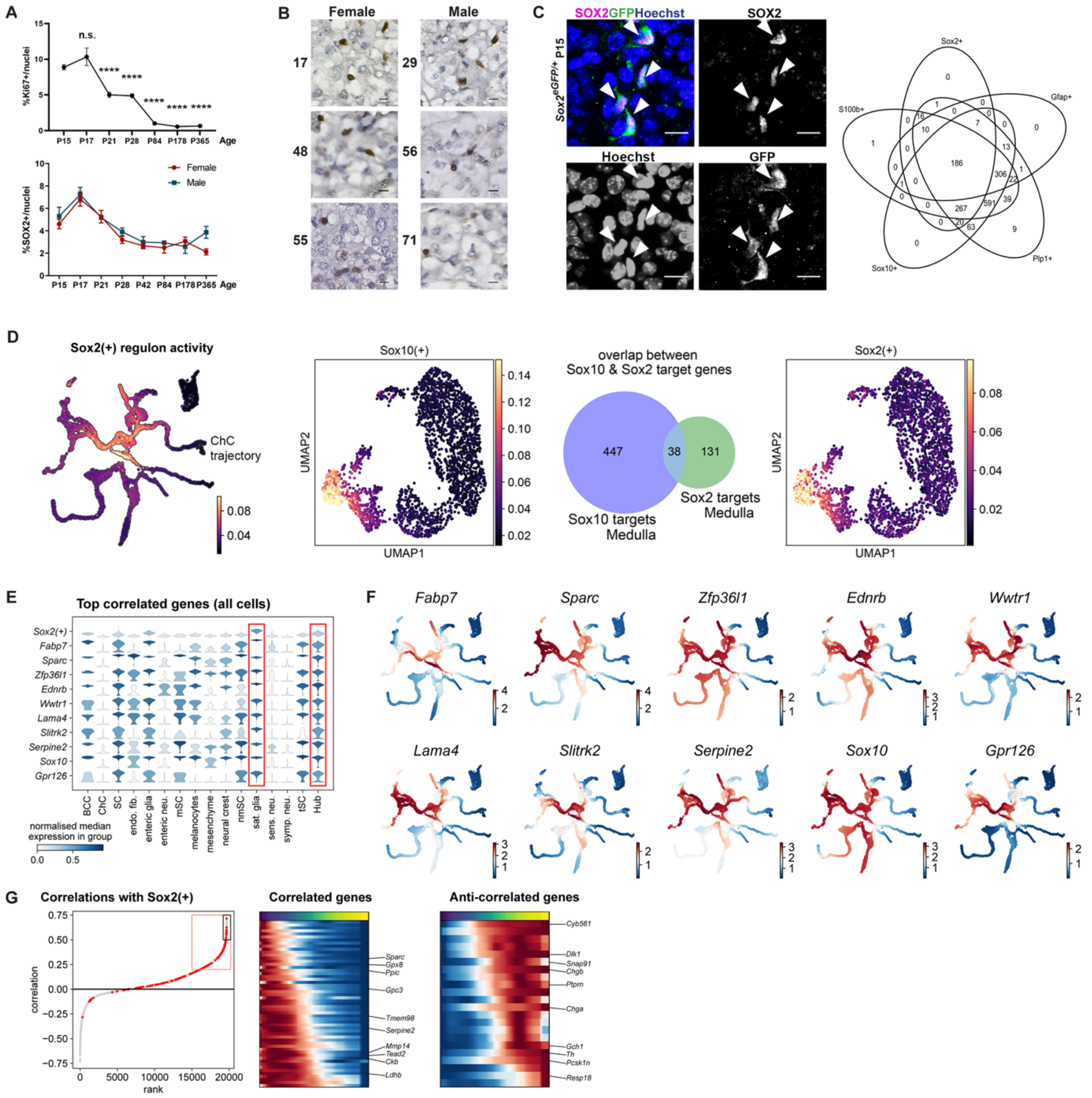

Supplementary Figure 2. SOX2⁺ cells are present in the mouse adrenal medulla and are derived from Schwann cell precursors. A) Quantification of Ki-67⁺ cells over the total nuclei of the adrenal medulla, *n* = 6 animals per time point, mean and SEM plotted. One-way ANOVA multiple comparisons test: P15 vs. P17 (*P*-value 0.1874); P15 vs. P21 (*P*-value <0.0001); P15 vs. P28 (*P*-value <0.0001); P15 vs. P42 (*P*-value <0.0001); P15 vs. P84 (*P*-value <0.0001); P15 vs. P178 (*P*-value <0.0001); P15 vs. P365 (*P*-value <0.0001). Quantification of SOX2⁺ cells over the total nuclei of adrenal medulla, split by sex. n = 3 animals per sex, per timepoint. Mean and SEM plotted. B) Immunohistochemistry with antibodies

against SOX2 (brown) on normal human adrenals in females (at 17, 48 and 55 years of age) and males (at 29, 56, and 71 years of age). Nuclei counterstained with Hematoxylin, scale bar 20µm. C) Immunofluorescence on *Sox2*$^{eGFP/+}$ adrenal medulla at P15, using antibodies against GFP (green) and SOX2 (magenta) showing complete co-localisation (arrowheads). Nuclei counterstained with Hoechst, scale bars 10µm. Venn diagram showing the overlap in adrenomedullary cells expressing markers *Sox2*, *Sox10*, *S100b*, *Gfap* and *Plp1*. D) Activity of the *Sox2* regulon in data from [22], showing this is active in the early part of the chromaffin cell trajectory (labelled). Activity of the *Sox2* and *Sox10* regulon in the postnatal dataset, Venn diagram showing the overlap of 38 genes from 447 *Sox10* target genes and 131 *Sox2* target genes. Featureplot colour scale represents regulon activity from low (black) to high (yellow). E) Top correlated genes to *Sox2*, irrespective of trajectory, which include several markers of the postnatal *Sox2*-expressing population. The highest expression is observed in satellite glia and Hub cells (red boxes). F) Featureplots of *Sox2*-correlated genes expression. Featureplot colour scale represents Log-normalised expression from low (blue) to high (red). G) Ranked correlation analysis identifying genes highest correlated with *Sox2* specifically in the chromaffin cell trajectory, and anti-correlated with *Sox2*, which includes chromaffin cell markers (*Th*, *Chga*, *Chgb*).

Supplementary Figure 3

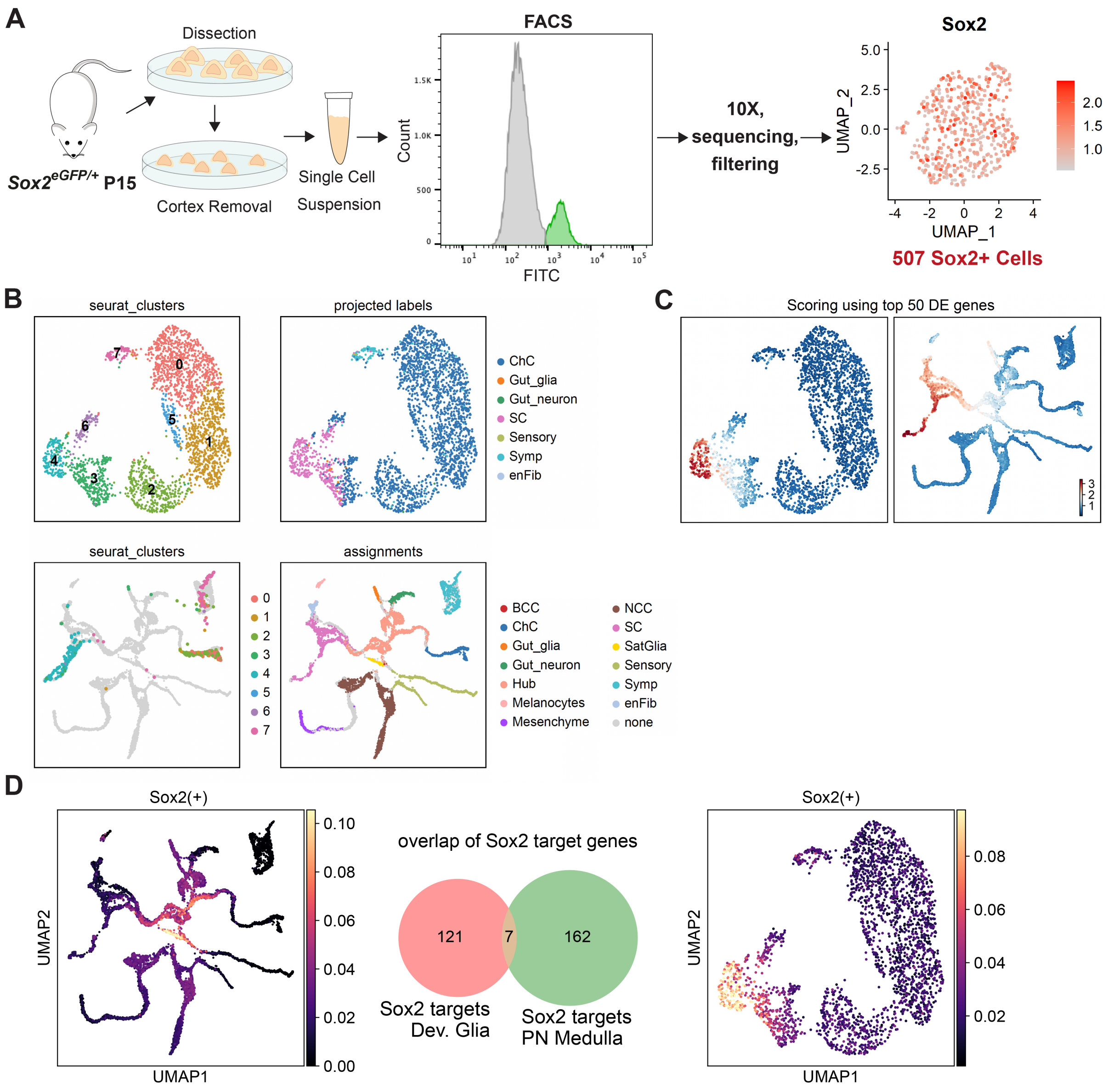

Supplementary Figure 3. Adrenomedullary SOX2^{+} stem cells are a distinct population from Schwann Cell Precursors. A) Experimental design. Featureplot showing *Sox2* expression in the selected dataset. Featureplot colour scale represents Log-normalised expression from low (grey) to high (red). B) Alignment of the postnatal dataset to the developmental glial dataset showing sustentacular cells mostly correlate with a postnatal Schwann cell identity. C) Top 50 differentially expressed genes from the *Sox2*-expressing sustentacular cluster scored on both postnatal and developmental datasets. Featureplot colour scale represents expression from low (blue) to high (red). D) Comparison of the

Sox2 regulon activity during development and postnatally, Venn diagram showing the overlap of 7 genes from 121 developmental target genes and 162 postnatal target genes. Featureplot colour scale represents regulon activity from low (black) to high (yellow).

Supplementary Figure 4

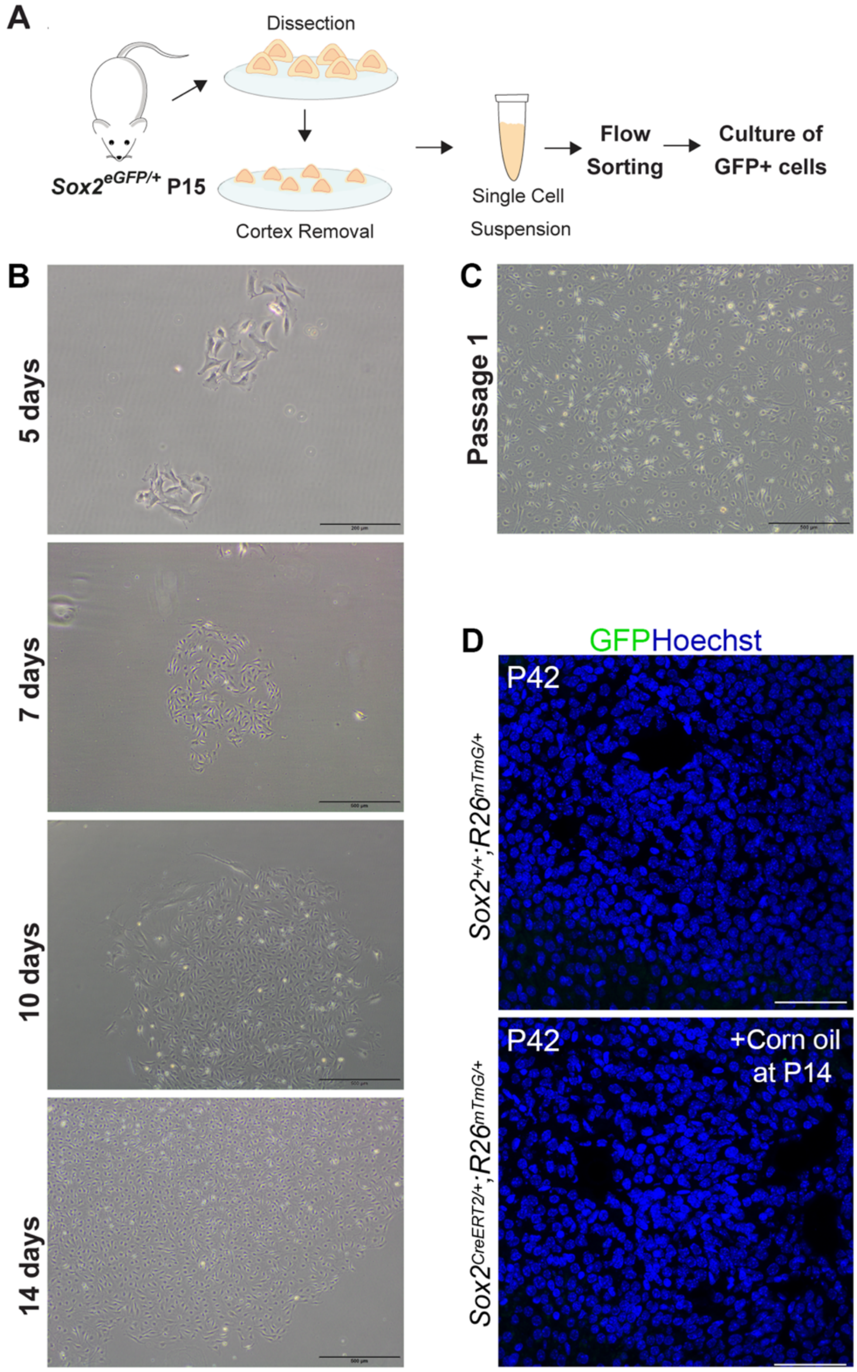

Supplementary Figure 4. Adrenomedullary SOX2^{+} cells have stem cell properties. A) Experimental design. B) Brightfield images of cultured GFP+ cells: 5, 7, 10, 14 days after isolation. Scale bars 200µm (5 days) and 500µm (7, 10, 14 days). C) Brightfield image of cultured GFP+ cells after 1 passage, scale bar 500µm. D) Immunofluorescence using antibodies against GFP (green) on sections from a

Sox2$^{+/+}$*;R26*$^{mTmG/+}$ adrenal at P42 (top panel) or *Sox2*$^{CreERT2/+}$*;R26*$^{mTmG/+}$ adrenal from a mouse injected with corn oil P14 and collected after 28 days (P42, bottom panel). Note the absence of GFP-labelled cells in these controls. Nuclei counterstained with Hoechst (blue), scale bar 50µm.

Supplementary Figure 5

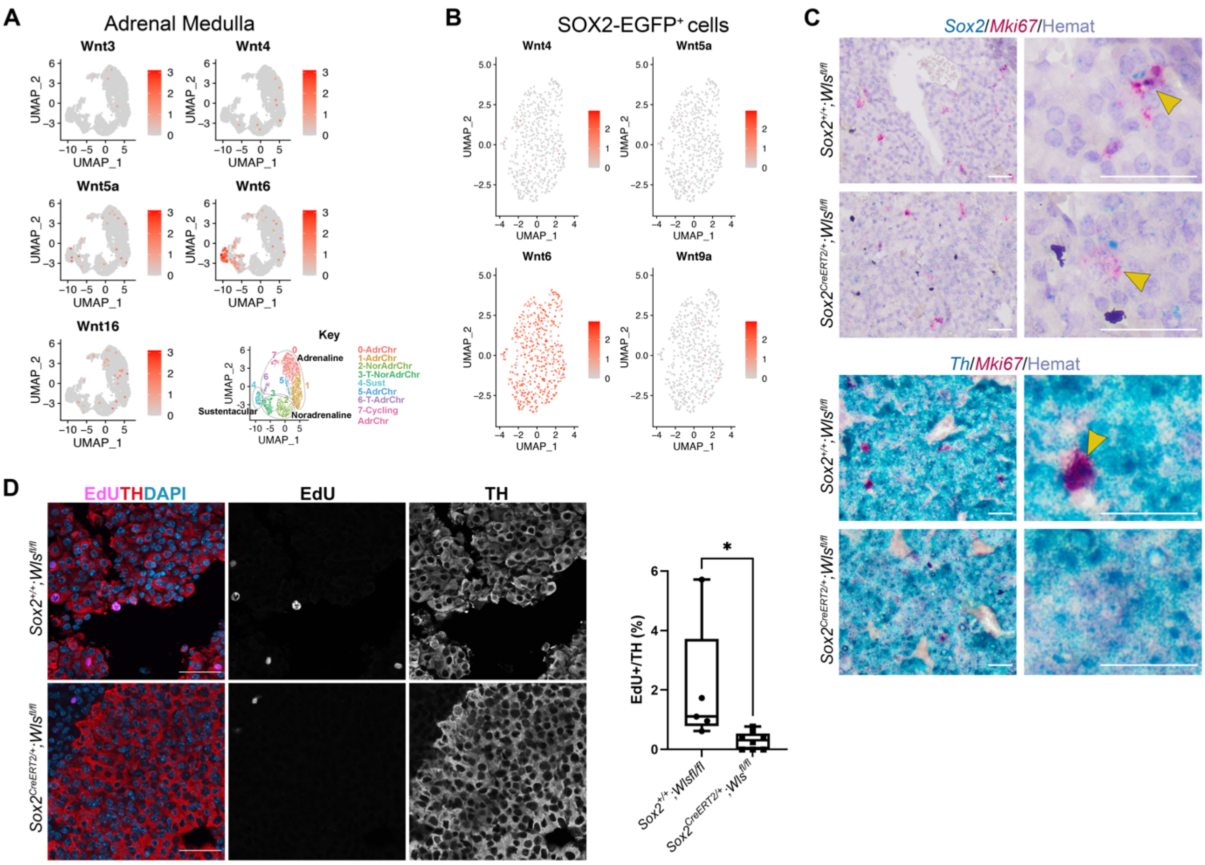

Supplementary Figure 5. SOX2+ adrenomedullary stem cells promote proliferation of chromaffin cells through secretion of paracrine WNT ligands. A) Featureplots for *Wnt3*, *Wnt4*, *Wnt5a*, *Wnt6*, and *Wnt16* in the mouse adrenal medulla dataset. Featureplot colour scale represents Log-normalised expression from low (grey) to high (red). B) Featureplots for *Wnt4*, *Wnt5a*, *Wnt6*, and *Wnt9a* in the isolated SOX2-EGFP^{+} cell dataset. Featureplot colour scale represents Log-normalised expression from low (grey) to high (red). C) RNAscope mRNA *in situ* hybridisation using probes against stem cell marker *Sox2* (blue) and cell cycle marker *Mki67* (red) or chromaffin marker *Th* (blue) and *Mki67* (red). Nuclei counterstained with hematoxylin. Yellow arrowheads indicate cells expressing both markers. Scale bars 25µm. D) Immunofluorescence staining using antibodies against TH (chromaffin cells) and EdU (cells in S-phase, detected using the Click-IT detection method) in *Sox2*$^{+/+}$;*Wls*$^{fl/fl}$ (control) and *Sox2*$^{CreERT2/+}$;*Wls*$^{fl/fl}$ (mutant) samples following tamoxifen induction at P13/P14/P15, a 2h EdU pulse and analysis at P21 (*n*=3 controls, 3 mutants). Nuclei counterstained with DAPI, scale bars 50µm.

Graph showing percentage of EdU positive cells as a percentage of TH positive cells, revealing a statistically significant reduction in cells in S-phase in the mutant (1.66% in control compared to 0.287% in mutant). Two-sided unpaired *t*-test, *P*-value = 0.0384. Source data are provided as a Source Data File.

Supplementary Table 1

REAGENT or RESOURCE	SOURCE	IDENTIFIER	Lot number	Publically available validation
Antibodies and immunofluorescence detection				
Anti-GFAP Rabbit polyclonal 1:500	Dako	Cat#Z0334	00059585	https://scicrunch.org/ResourceWatch/Search?q=AB_10013382
Anti-GFAP Chicken polyclonal 1:1000 x	Antibodies.com	Cat#A85307	X529	https://www.antibodies.com/gfap-antibody-a85307
Anti-GFP Chicken polyclonal 1:300 x	Abcam	Cat#ab13970	GR236651-12	https://www.abcam.com/en-us/products/primary-antibodies/gfp-antibody-ab13970
Anti-PENK Rabbit polyclonal 1:300 x	ABclonal Technology	Cat#A6302	5500018513	https://static.abclonal.com/datasheet/A6302.pdf
Anti-PNMT Mouse monoclonal 1:300 x	Santa Cruz	Cat#sc-393995	B1120	https://www.scbt.com/p/pnmtase-antibody-c-7?srsltid=AfmBOooeH3JT45xeejSaYi2Ubc4vVTPQuAcheaWrobPUwEioltaqKeom
Anti-S100b (EP1576Y) Rabbit monoclonal 1:300 x	Abcam	Cat#ab52642	GR296650-27	https://www.abcam.com/en-us/products/primary-antibodies/s100-beta-antibody-ep1576y-astrocyte-marker-ab52642
Anti-SOX10 (SD204-04) Rabbit monoclonal 1:100 xx	Novus	Cat#NBP2-67812	H00118	https://www.novusbio.com/PDFs4/NBP2-67812.pdf

Anti-SOX2 (EPR3131) Rabbit Monoclonal 1:300 xx	Abcam	Cat#ab92494	1007374-4	https://www.abcam.com/en-us/products/primary-antibodies/sox2-antibody-epr3131-ab92494#
Anti-Tyrosine Hydroxylase Mouse Monoclonal 1:300 xx	BD Biosciences	Cat#612300	2301592	https://www.citeab.com/antibodies/2411748-612300-bd-transduction-laboratories-purified-mouse
Anti-Chicken Alexa Fluor 488 Goat polyclonal 1:500 xx	Invitrogen	Cat#A-11039	2566343	https://www.thermofisher.com/antibody/product/Goat-anti-Chicken-IgY-H-L-Secondary-Antibody-Polyclonal/A-11039
Anti-Mouse Biotinylated Goat polyclonal 1:300 xx	Abcam	Cat#ab6788	GR3213189-2	https://www.abcam.com/en-us/products/secondary-antibodies/goat-mouse-igg-h-l-biotin-ab6788
Anti-Rabbit Alexa Fluor 488 Goat polyclonal 1:500 xx	Invitrogen	Cat#A-11008	2284595	https://www.thermofisher.com/antibody/product/Goat-anti-Rabbit-IgG-H-L-Cross-Adsorbed-Secondary-Antibody-Polyclonal/A-11008

Anti-Rabbit Alexa Fluor 594 Goat polyclonal 1:500	Abcam	Cat#ab150080	GR3440097-1	https://www.abcam.com/en-us/products/secondary-antibodies/goat-rabbit-igg-h-l-alexa-fluor-594-ab150080
Streptavidin Alexa Fluor 555	Invitrogen	Cat#S32355	2031434	https://www.thermofisher.com/order/catalog/product/S32355?SID=srch-hj-S32355
Streptavidin Alexa Fluor 488	Invitrogen	Cat#S11223	1445277	https://www.thermofisher.com/order/catalog/product/S11223
Click-iT EdU Cell Proliferation Kit for Imaging, Alexa Fluor 647 dye	Invitrogen	Cat#C10340	2031166	https://www.thermofisher.com/order/catalog/product/C10340
Biological samples				
Normal human adrenal	University Hospital Würzburg	European Network for Adrenal Tumours ENS@T		
Chemicals, peptides, and recombinant proteins				
Tamoxifen	Sigma	Cat#T5648		
Progesterone	Sigma	Cat#P0130		
Neutral Buffered Formalin	Sigma	Cat#HT501128		
Hematoxylin QS	Vector Laboratories	Cat#H-3404-100		
VectaMount Permanent Mounting Medium	Vector Laboratories	Cat#H-500-60		
Vectashield Antifade Mounting Medium	Vector Laboratories	Cat#H-1000-10		
Vectashield HardSet Antifade Mounting Medium with DAPI	Vector Laboratories	Cat#H-1500-10		
Hoechst 33258, pentahydrate (bis-benzimide) 10mg/ml	Invitrogen	Cat#H3569		
DNAse I	Sigma	Cat#D5025		
Collagenase II	Worthington	Cat#LS004177		
Fungizone	Gibco	Cat#15290026		
Trypsin-EDTA	Sigma	Cat#59418C		
DMEM/F-12	Gibco	Cat#31330-038		
Fetal Bovine Serum	Merk	Cat#F0804		
Pen-Strep	Gibco	Cat#15070063		
bFGF	R&D Systems	Cat#234-FSE		
Cholera toxin	Sigma	Cat#C8052		
EdU (5-ethynyl-2′-deoxyuridine)	Invitrogen	Cat#A10044		
Critical commercial assays				

RNAscope 2.5 HD Duplex Kit	ACD Bio	Cat#322430		
ImmPRESS Excel Amplified HRP Polymer Staining Kit Anti-Rabbit IgG	Vector Laboratories	Cat#MP-7602-50		
Chromium Next GEM Single Cell 3' GEM, Library & Gel Bead Kit v3.1	10x Genomics	Cat#PN-1000121		
Deposited data				
This paper datasets		GEO accession number: GSE237125		
Developmental Dataset from Kastriti *et al.*	https://www.embopress.org/doi/full/10.15252/embj.2021108780	GEO accession number: GSE201257		
Code	GitHub	https://github.com/Andoniadou-Lab/adrenal_stemcell		
Experimental models: Organisms/strains				
$Sox2^{eGFP/+}$	Gifted from Larysa Pevny Ellis et al., 2004 PMID:15711057 DOI: 10.1159/000082134	MGI:3589809		
$Sox2^{CreERT2/+}$	Generated in house *Andoniadou et al., 2013* PMID:24094324 DOI: 10.1016/j.stem.2013.07.004	MGI:5512893		
$Wnt1^{Cre/+}$	Gifted from Karen Liu			
$Sox10^{iCreERT2/+}$	The Jackson Laboratory	Cat#027651		
$R26^{mTmG/mTmG}$	The Jackson Laboratory	Cat#007576		
$Wls^{flox/flox}$	The Jackson Laboratory	Cat#012888		
Shaver Brown eggs	Medeggs Ltd			
Oligonucleotides				
Generic Cre ($Sox2^{CreERT2}$, $Wnt1^{Cre}$) Primer 1	Sigma	GAT GCA ACG AGT GAT GAG GTT CGC		
Generic Cre ($Sox2^{CreERT2}$, $Wnt1^{Cre}$) Primer 2	Sigma	ACC CTG ATC CTG GCA ATT TCG GC		
$R26^{mTmG}$ Primer 1	Sigma	CTC TGC TGC CTC CTG GCT TCT		
$R26^{mTmG}$ Primer 2	Sigma	CGA GGC GGA TCA CAA GCA ATA		
$R26^{mTmG}$ Primer 3	Sigma	TCA ATG GGC GGG GGT CGT T		
$Sox10^{iCreERT2}$ Primer 1	Sigma	TGC CCA GAG TCA TCC TTG GC		
$Sox10^{iCreERT2}$ Primer 2	Sigma	GAG GGA CTA CCT CCT GTA CC		
$Sox2^{eGFP}$ Primer 1	Sigma	CGC TTC CTC GTG CTT TAC G		
$Sox2^{eGFP}$ Primer 2	Sigma	GGC TTC TCC TTT TTT TGC AGT		

Wls^{flox} Primer 1	Sigma	AGG CTT CGA ACG TAA CTG ACC		
Wls^{flox} Primer 2	Sigma	CTC AGA ACT CCC TTC TTG AAG C		
Plp1 RNAscope probe	ACD Bio	Cat#428181		
S100b-C2 RNAscope probe	ACD Bio	Cat#431731-C2		
Sox10 RNAscope probe	ACD Bio	Cat#435931		
Sox2 RNAscope probe	ACD Bio	Cat#401041		
Sox2-C2 RNAscope probe	ACD Bio	Cat#401041-C2		
Th RNAscope probe	ACD Bio	Cat#317621		
Wnt6 RNAscope probe	ACD Bio	Cat#401111		
MKi67 RNAscope probe	ACD Bio	Cat#416771		
MKi67-C2 RNAscope probe	ACD Bio	Cat#416771-C2		
Ppib +ve CTRL RNAscope probe	ACD Bio	Cat#313911		
DapB -ve *CTRL* RNAscope probe	ACD Bio	Cat#310043		
Software and algorithms				
GraphPad Prism	GraphPad	https://www.graphpad.com/		
Zen Blue	Zeiss			
LeicaTCS SP5	Leica			
Fiji		https://imagej.net/software/fiji/		
Nanozoomer Digital Pathology View	Hamamatsu			
Illustrator	Adobe	https://www.adobe.com/uk/products/illustrator		
Rstudio		https://posit.co/products/open-source/rstudio/		
Seurat		https://satijalab.org/seurat/		
Tidyverse		https://www.tidyverse.org/		
Monocle3		https://cole-trapnell-lab.github.io/monocle3/		
ClusterProfiler		https://bioconductor.org/packages/release/bioc/html/clusterProfiler.html		
scFates 1.0.8		https://github.com/LouisFaure/scFates		
CONOS R		https://github.com/kharchenkolab/conos		
SCENIC		https://github.com/aertslab/SCENIC		

179
