# Supplementary material for: SOX2+ sustentacular cells are stem cells of the postnatal adrenal medulla
Source: Nat Commun. 2025 Jan 2;16:16. doi: 10.1038/s41467-024-55289-5 (PMC11696870; doi:10.1038/s41467-024-55289-5)
Supplement: Supplementary file 3 — Description of Additional Supplementary Files [file 41467_2024_55289_MOESM3_ESM.pdf]

Description of Additional Supplementary Files

Supplementary Data 1 - SCENIC analysis comparing Sox2 and Sox10 regulons during the chromaffin commitment trajectory. Target genes corresponding to the ‘Sox2’ or the ‘Sox10’ regulon are listed, as indicated in the list in the column TF for ‘transcription factor’. The majority of Sox2 and Sox10 targets are distinct (Sox10-only 447, Sox2-only 131, shared 38).

Supplementary Data 2 - SCENIC analysis comparing Sox2 regulons in developmental glia and in cells of the postnatal medulla. Target genes corresponding to the ‘Sox2 (Dev. Glia)’ or the ‘Sox2 (Medulla)’ regulon are listed, as indicated in the list in the column TF for ‘transcription factor’. There is little overlap between Sox2 targets, with 121 unique Sox2 targets in developmental glia, 162 Sox2 targets in the postnatal medulla, and 7 shared targets.
