# Supplementary material for: SOX2+ sustentacular cells are stem cells of the postnatal adrenal medulla
Source: Nat Commun. 2025 Jan 2;16:16. doi: 10.1038/s41467-024-55289-5 (PMC11696870; doi:10.1038/s41467-024-55289-5)
Supplement: Supplementary file 6 — Reporting Summary [file 41467_2024_55289_MOESM6_ESM.pdf]

nature portfolio

Corresponding author(s): Cynthia Andoniadou

Last updated by author(s): Jul 23, 2024

Reporting Summary

Nature Portfolio wishes to improve the reproducibility of the work that we publish. This form provides structure for consistency and transparency in reporting. For further information on Nature Portfolio policies, see our Editorial Policies and the Editorial Policy Checklist.

Statistics

For all statistical analyses, confirm that the following items are present in the figure legend, table legend, main text, or Methods section.

n/a	Confirmed	
☐	☒	The exact sample size (*n*) for each experimental group/condition, given as a discrete number and unit of measurement
☐	☒	A statement on whether measurements were taken from distinct samples or whether the same sample was measured repeatedly
☐	☒	The statistical test(s) used AND whether they are one- or two-sided<br>*Only common tests should be described solely by name; describe more complex techniques in the Methods section.*
☐	☒	A description of all covariates tested
☐	☒	A description of any assumptions or corrections, such as tests of normality and adjustment for multiple comparisons
☐	☒	A full description of the statistical parameters including central tendency (e.g. means) or other basic estimates (e.g. regression coefficient) AND variation (e.g. standard deviation) or associated estimates of uncertainty (e.g. confidence intervals)
☐	☒	For null hypothesis testing, the test statistic (e.g. F, t, r) with confidence intervals, effect sizes, degrees of freedom and P value noted<br>*Give P values as exact values whenever suitable.*
☐	☒	For Bayesian analysis, information on the choice of priors and Markov chain Monte Carlo settings
☐	☒	For hierarchical and complex designs, identification of the appropriate level for tests and full reporting of outcomes
☐	☒	Estimates of effect sizes (e.g. Cohen's *d*, Pearson's *r*), indicating how they were calculated

Our web collection on statistics for biologists contains articles on many of the points above.

Software and code

Policy information about availability of computer code

Data collection	Code available at: https://github.com/Andoniadou-Lab/adrenal_stemcell.
Data analysis	Analysis tools used: Cell Ranger 4.0.0 (https://www.10xgenomics.com/support/software/cell-ranger/latest), Rstudio (https://posit.co/products/open-source/rstudio/), Seurat v3 and v4 (https://satijalab.org/seurat/), Tidyverse (https://www.tidyverse.org/), Monocle3 (https://cole-trapnell-lab.github.io/monocle3/), ClusterProfiler (https://bioconductor.org/packages/release/bioc/html/clusterProfiler.html), scFates 1.0.8 (https://github.com/LouisFaure/scFates), CONOS R (https://github.com/kharchenkolab/conos), SCENIC (https://github.com/aertslab/SCENIC).

For manuscripts utilizing custom algorithms or software that are central to the research but not yet described in published literature, software must be made available to editors and reviewers. We strongly encourage code deposition in a community repository (e.g. GitHub). See the Nature Portfolio guidelines for submitting code & software for further information.

Data

Policy information about availability of data

All manuscripts must include a data availability statement. This statement should provide the following information, where applicable:

- Accession codes, unique identifiers, or web links for publicly available datasets
- A description of any restrictions on data availability
- For clinical datasets or third party data, please ensure that the statement adheres to our policy

Datasets are available at the Gene Expression Omnibus (GEO) with accession number GSE237125.

Research involving human participants, their data, or biological material

Policy information about studies with human participants or human data. See also policy information about sex, gender (identity/presentation), and sexual orientation and race, ethnicity and racism.

Reporting on sex and gender	Sex was considered for the samples used in this study to qualitatively report on the presence or absence of a particular cell type (SOX2+ cells). No differences were observed across sexes.
Reporting on race, ethnicity, or other socially relevant groupings	These data were not available on the samples sourced.
Population characteristics	Age and sex were reported for the samples used in this study.
Recruitment	All samples available to us (6 biological samples) were included, spanning a range of ages (17-71 years) and across both sexes.
Ethics oversight	Studies using human adrenals were carried out under King's College London ethical approval with KCL Ethics Reference LRS-19/20-20118, samples were sourced as part of the adrenal tumour registry project of the European Network for Adrenal Tumours ENS@T (European Network for the Study of Adrenal Tumours).

Note that full information on the approval of the study protocol must also be provided in the manuscript.

Field-specific reporting

Please select the one below that is the best fit for your research. If you are not sure, read the appropriate sections before making your selection.

☒ Life sciences ☐ Behavioural & social sciences ☐ Ecological, evolutionary & environmental sciences

For a reference copy of the document with all sections, see nature.com/documents/nr-reporting-summary-flat.pdf

Life sciences study design

All studies must disclose on these points even when the disclosure is negative.

Sample size	The Experimental Design Assistant by the NC3Rs was consulted to determine sample sizes (https://eda.nc3rs.org.uk/). We assume sex-specific differences in post-pubertal adrenals, therefore each experiment has four groups: control male and female, mutant male and female where both genotype and sex are considered factors of interest. We expected the data to fit parametric assumptions.
Data exclusions	No data were excluded from the analyses.
Replication	Experiments were replicated to ensure reproducibility. Where possible, findings have also been verified through independent experimental means i.e. using a different approach.
Randomization	Randomization was not relevant to the study. Where group comparisons are made these are between groups with different genotypes, where the genotype is known.
Blinding	For experiments necessitating counting and quantification, this was carried out independently by two researchers for each experiment. One of the researchers performed the quantification blind, where genotype and identifying labels had been removed.

Reporting for specific materials, systems and methods

We require information from authors about some types of materials, experimental systems and methods used in many studies. Here, indicate whether each material, system or method listed is relevant to your study. If you are not sure if a list item applies to your research, read the appropriate section before selecting a response.

Materials & experimental systems

n/a	Involved in the study
☐	☒ Antibodies
☒	☐ Eukaryotic cell lines
☒	☐ Palaeontology and archaeology
☐	☒ Animals and other organisms
☒	☐ Clinical data
☒	☐ Dual use research of concern
☒	☐ Plants

Methods

n/a	Involved in the study
☒	☐ ChIP-seq
☒	☐ Flow cytometry
☒	☐ MRI-based neuroimaging

Antibodies

Antibodies used	All antibodies used in this study are commercially available: Anti-GFAP Rabbit polyclonal 1:500 (Dako Cat#Z0334), Anti-GFAP Chicken polyclonal 1:1000 (Antibodies.com, Cat#A85307), Anti-GFP Chicken polyclonal 1:300 (Abcam Cat#ab13970), Anti-PENK Rabbit polyclonal 1:300 (ABclonal Technology Cat#A6302), Anti-PNMT Mouse monoclonal 1:300 (Santa Cruz Cat#sc-393995), Anti-S100b (EP1576Y) Rabbit monoclonal 1:300 (Abcam, Cat#ab52642), Anti-SOX10 (SD204-04) Rabbit monoclonal 1:100 (Novus Cat#NBP2-67812), Anti-SOX2 (EPR3131) Rabbit Monoclonal 1:300 (Abcam Cat#ab92494), Anti-Ki-67 Rat monoclonal 1:300 (Invitrogen Cat#14-5698-82), Anti-Tyrosine Hydroxylase Mouse Monoclonal 1:300 (BD Biosciences Cat#612300), Anti-Chicken Alexa Fluor 488 Goat polyclonal 1:500 (Invitrogen Cat#A-11039), Anti-Mouse Biotinylated Goat polyclonal 1:300 (Abcam Cat#ab6788), Anti-Rabbit Alexa Fluor 488 Goat polyclonal 1:500 (Invitrogen Cat#A-11008), Anti-Rabbit Alexa Fluor 594 Goat polyclonal 1:500 (Abcam Cat#ab150080).
Validation	All primary antibodies have validation data on the manufacturers websites. The link has been provided in the Key Resources Table. Prior to using each primary antibody in this study, we performed a no primary control. For antibodies against SOX2, SOX10, KI-67, TH, PNMT and PENK, we compared the protein expression patterns to mRNA expression using specific probes, ensuring overlap.

Animals and other research organisms

Policy information about studies involving animals; ARRIVE guidelines recommended for reporting animal research, and Sex and Gender in Research

Laboratory animals	Mice and fertilized chicken eggs were used. Mice: All mice were bred and maintained on CD1 background, except Wlsfl/fl, which was used on a mixed CD1 x C57BL/6 background and consistently backcrossed on CD1. Strains used were: Sox2eGFP/+, Sox2CreERT2/+, Wnt1Cre/+, Sox10iCreERT2/+, R26mTmG/+, Wlsfl/fl. Chicken: Fertilized Shaver Brown eggs were used. Animals were used at the following ages: P15 (single cell analysis, in vitro experiments, in ovo experiments), P15, P17, P21, P28, P42, P84, P178, P365 (analysis of SOX2+ cells), P17, P21, P28, P42, P84, P192, P217, P379 (lineage tracing).
Wild animals	The study did not involve wild animals.
Reporting on sex	We assumed sex-specific differences in post-pubertal adrenals, therefore analysing four groups: control male and female, mutant male and female where both genotype and sex are considered factors of interest. Data have been split by sex, shown in Figures S1D, FIgure S2A, Figure S2B. The analyses concluded that sex differences are not relevant to the study.
Field-collected samples	The study did not involve samples collected from the field.
Ethics oversight	All animal studies received KCL Biological Safety approval for project 'Function and Regulation of Adrenal Stem Cells in Mammals'. Experiments were performed under compliance of the Animals (Scientific Procedures) Act 1986, Home Office Licences P5F0A1579 (mouse) and P8D5E2773 (chicken).

Note that full information on the approval of the study protocol must also be provided in the manuscript.

Plants

Seed stocks	*Report on the source of all seed stocks or other plant material used. If applicable, state the seed stock centre and catalogue number. If plant specimens were collected from the field, describe the collection location, date and sampling procedures.*
Novel plant genotypes	*Describe the methods by which all novel plant genotypes were produced. This includes those generated by transgenic approaches, gene editing, chemical/radiation-based mutagenesis and hybridization. For transgenic lines, describe the transformation method, the number of independent lines analyzed and the generation upon which experiments were performed. For gene-edited lines, describe the editor used, the endogenous sequence targeted for editing, the targeting guide RNA sequence (if applicable) and how the editor was applied.*
Authentication	*Describe any authentication procedures for each seed stock used or novel genotype generated. Describe any experiments used to assess the effect of a mutation and, where applicable, how potential secondary effects (e.g. second site T-DNA insertions, mosiacism, off-target gene editing) were examined.*
